# Supplementary material for: HIV-phyloTSI: subtype-independent estimation of time since HIV-1 infection for cross-sectional measures of population incidence using deep sequence data
Source: BMC Bioinformatics. 2025 Aug 14;26:212. doi: 10.1186/s12859-025-06189-y (PMC12351810; doi:10.1186/s12859-025-06189-y)
Supplement: Supplementary file 1 — Supplementary Material 1. [file 12859_2025_6189_MOESM1_ESM.docx]

Supplementary Materials for

**HIV-phyloTSI: Subtype-independent estimation of time since HIV-1 infection for cross-sectional measures of population incidence using deep sequence data**

Tanya Golubchik^1,44^*, Lucie Abeler-Dörner^1^, Matthew Hall^1^, Chris Wymant^1^, David Bonsall^1,2^, George Macintyre-Cockett^1,2^, Laura Thomson^1^,  Jared M. Baeten^3^, Connie L Celum^3-5^, Ronald M. Galiwango^6^, Barry Kosloff^7,8^, Mohammed Limbada^7,8^, Andrew Mujugira^9^, Nelly R Mugo^3,10^, Astrid Gall^11^, François Blanquart^12,13^, Margreet Bakker^14^, Daniela Bezemer^15^, Swee Hoe Ong^16^, Jan Albert^17,18^, Norbert Bannert^19^, Jacques Fellay^20-22^, Barbara Gunsenheimer-Bartmeyer^23^, Huldrych F. Günthard^24,25^, Pia Kivelä^26^, Roger D. Kouyos^24,25^, Laurence Meyer^27^, Kholoud Porter^28^, Ard van Sighem^15^, Mark van der Valk^29^, Ben Berkhout^14^,  Paul Kellam^30^, Marion Cornelissen^14^, Peter Reiss^15,31^, Helen Ayles^7,8^, David N. Burns^32^, Sarah Fidler^33^, Mary Kate Grabowski^34,35^,  Richard Hayes^7^, Joshua T Herbeck^36^, Joseph Kagaayi^6^, Pontiano Kaleebu^37-39^, Jairam R Lingappa^3,4,40^, Deogratius Ssemwanga^37-39^, Susan H Eshleman^35^, Myron S Cohen^41^, Oliver Ratmann^42^, Oliver Laeyendecker^43^, Christophe Fraser^1^ on behalf of the HPTN 071 (PopART) Phylogenetics protocol team, the BEEHIVE consortium and the PANGEA consortium.

*Corresponding author. Email: tanya.golubchik@sydney.edu.au

**This PDF file includes:**

Supplementary

Figs. S1 to S9

Tables S1 to S3


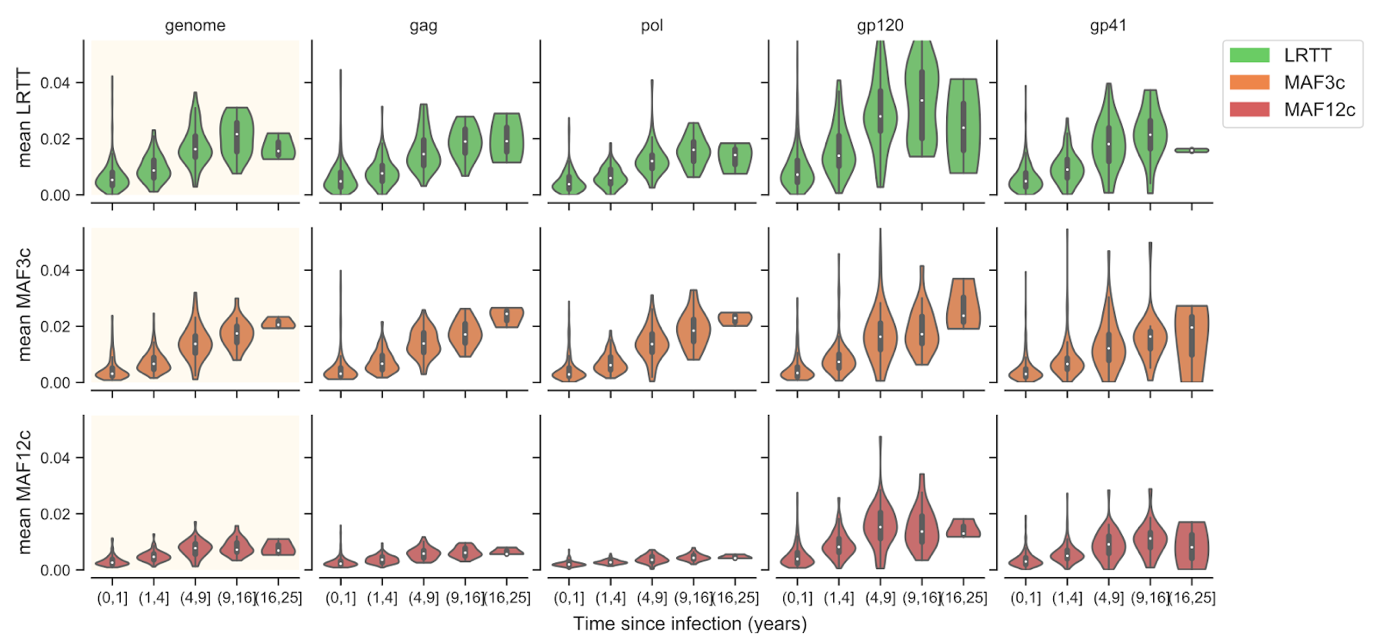


Fig. S1.
Genetic divergence (LRTT) and genetic diversity (MAF3c, MAF2c) for individual windows plateau at longer duration of infection. Vertical bars indicate 95% bootstrap confidence interval for the mean of each predictor, aggregated across the entire genome (first column, shaded) or within each of *gag*, *pol*, *gp120* and *gp41* HIV genes, for all samples.

**
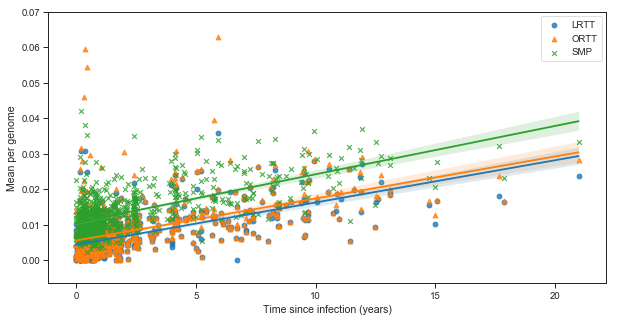
**

Fig. S2.

**Phylogenetic metrics reported by phyloscanner are correlated with TSI.** LRTT, largest subgraph root-to-tip distance; ORTT, overall root-to-tip distance; SMP, subgraph mean patristic distance. All three metrics are corrected with TSI, and are strongly correlated with one another, with some noisier estimates particularly for ORTT where a sample had multiple subgraphs due to e.g., contaminating reads in the window. For this analysis LLRT was selected, as this metric is most robust to presence of multiple subgraphs (i.e., individuals infected with multiple viral strains).


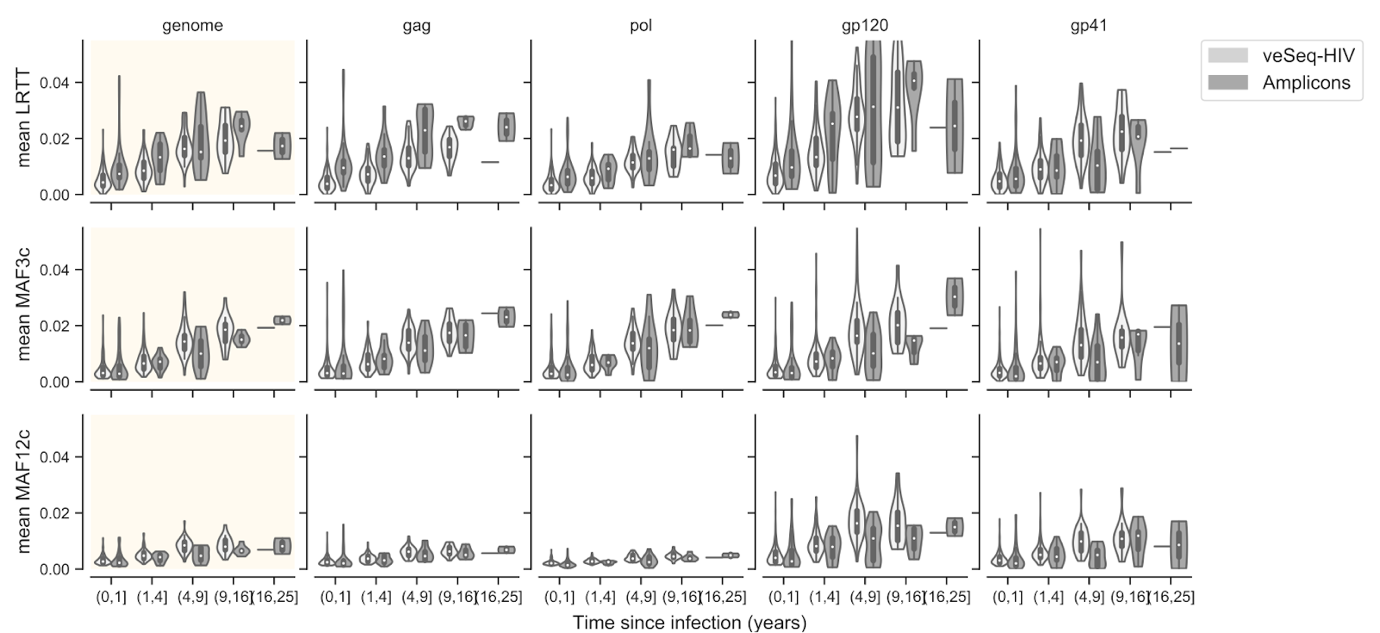


Fig. S3.

**Mean divergence (LRTT) and diversity (MAF3c, MAF12c) measures increase with duration of infection in both veSeq-HIV and amplicon sequence data.** Vertical bars indicate 95% bootstrap confidence interval for the mean of each predictor, aggregated across the entire genome (first column, shaded axes) or within each of gag, pol, gp120 and gp41 HIV genes, separately for veSeq-HIV sequences (light violins) and amplicon sequences (dark violins).


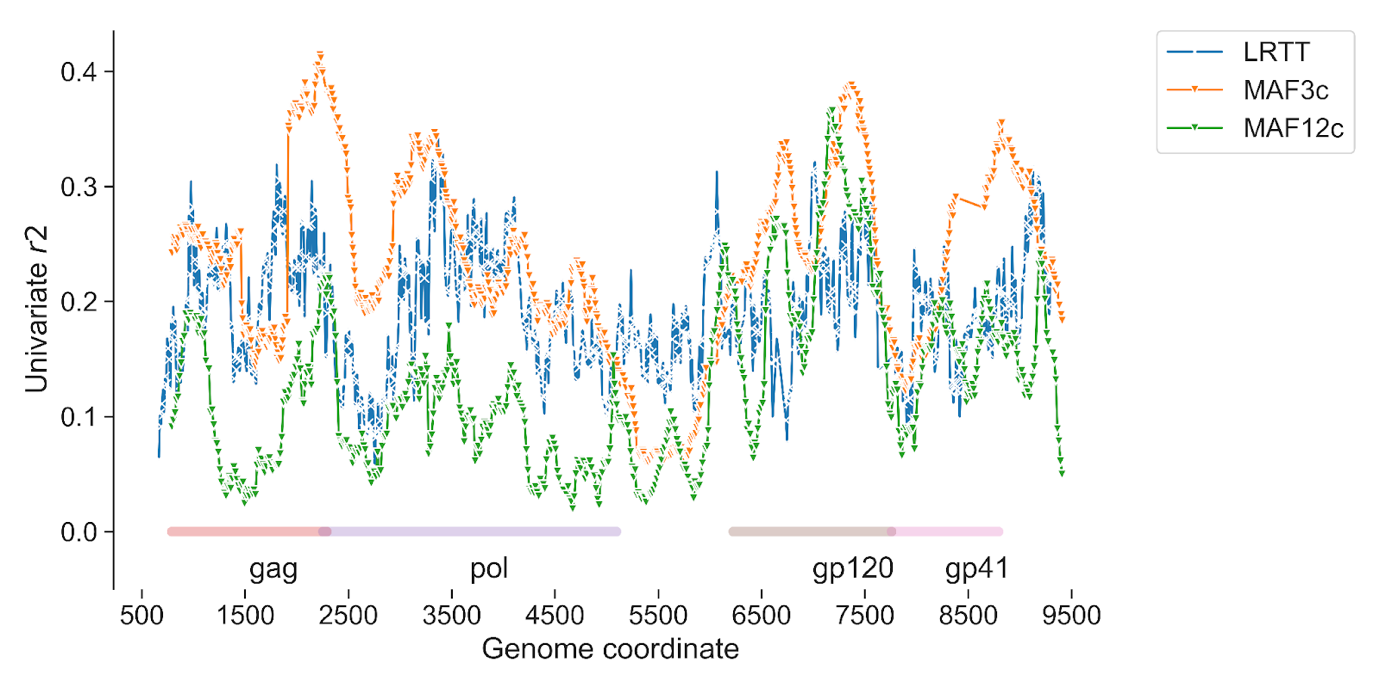


Fig. S4.

**Correlations with TSI for predictors across the HIV-1 genome.** Univariate linear regression models (OLS) were fitted independently for LRTT, MAF3c and MAF12c in overlapping 250b genomic windows, using the square root of the estimated duration of infection as the target variable. Missing data were zero-filled. Shown is the r^2^ within each window, with the window centre plotted on the x-axis.


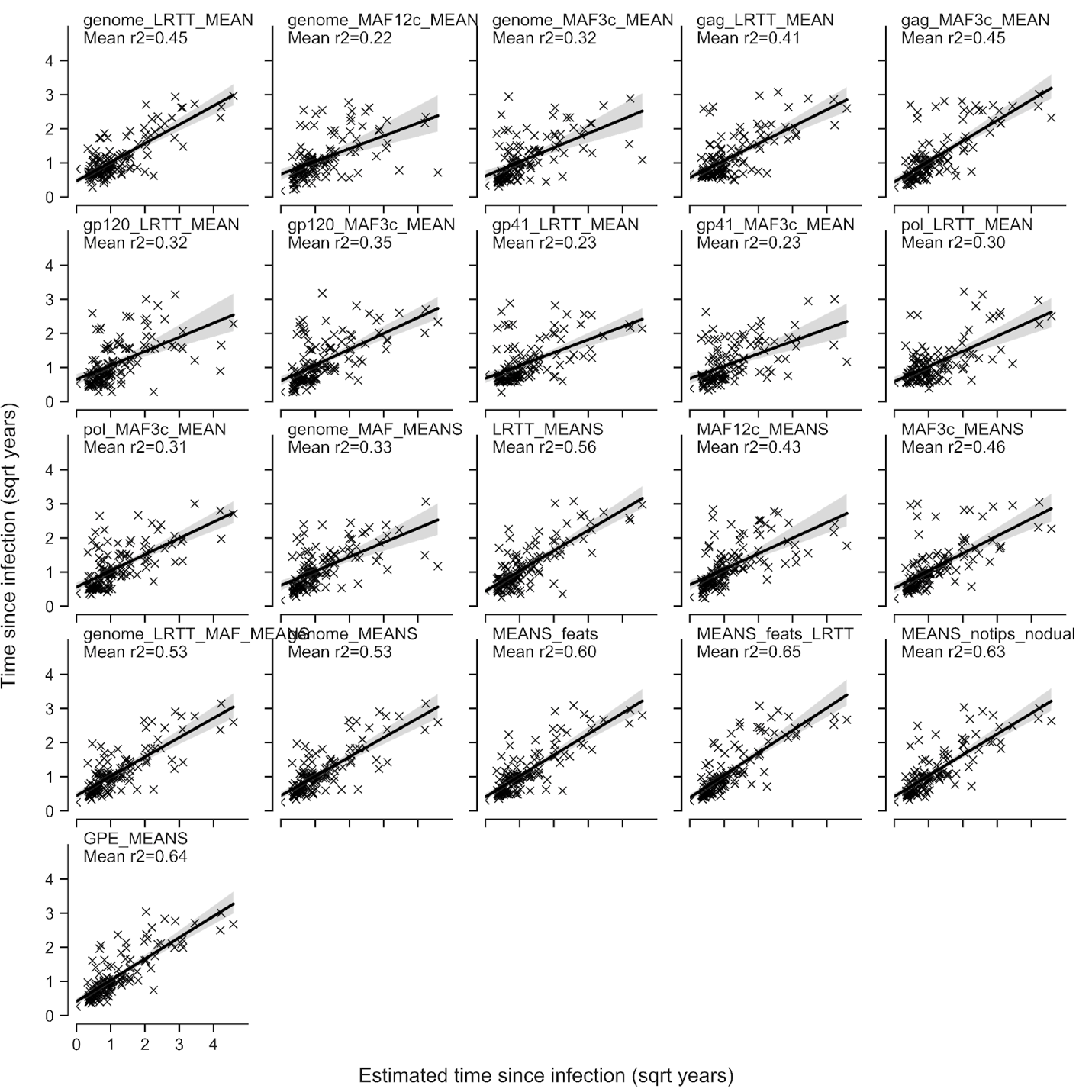


Fig. S5.

**Scatterplots of known versus estimated square root-transformed time since infection for aggregate regression models in Table S2, derived using random forest regression.** Models are named in correspondence with Table S2. Shown are scatterplots for the same set of training:test data (fold) for all models.


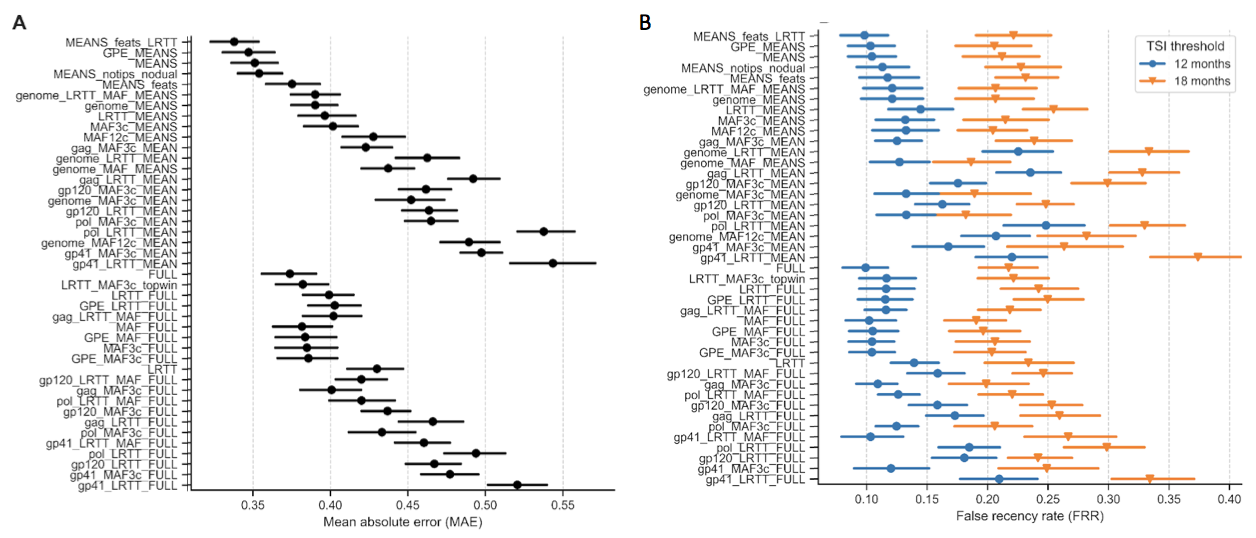


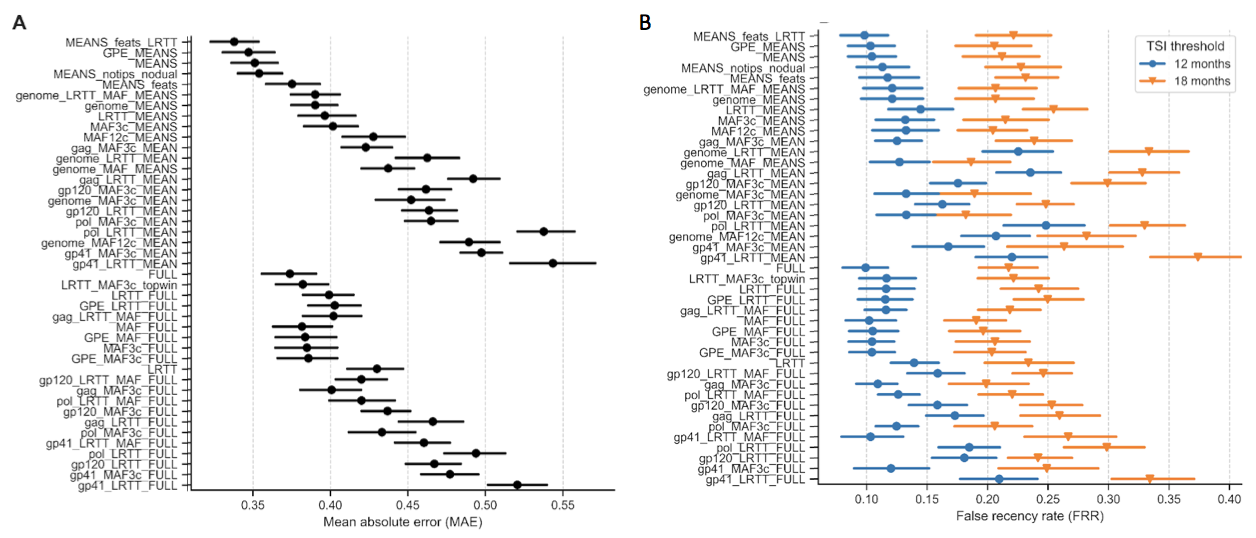


Fig. S6.

**Mean absolute error (MAE) and estimated false recency rate (FRR).** In all panels, circles show mean and lines show 95% bootstrap CI over 20-fold cross-validation. A. MAE, calculated as absolute difference between known and estimated TSI in square-root space. B. D. FRR, computed as the fraction of samples with known TSI over 12 months (circles, blue) or 18 months (triangles, orange) for which TSI was incorrectly estimated as being below 12 or 18 months, respectively.

**A**


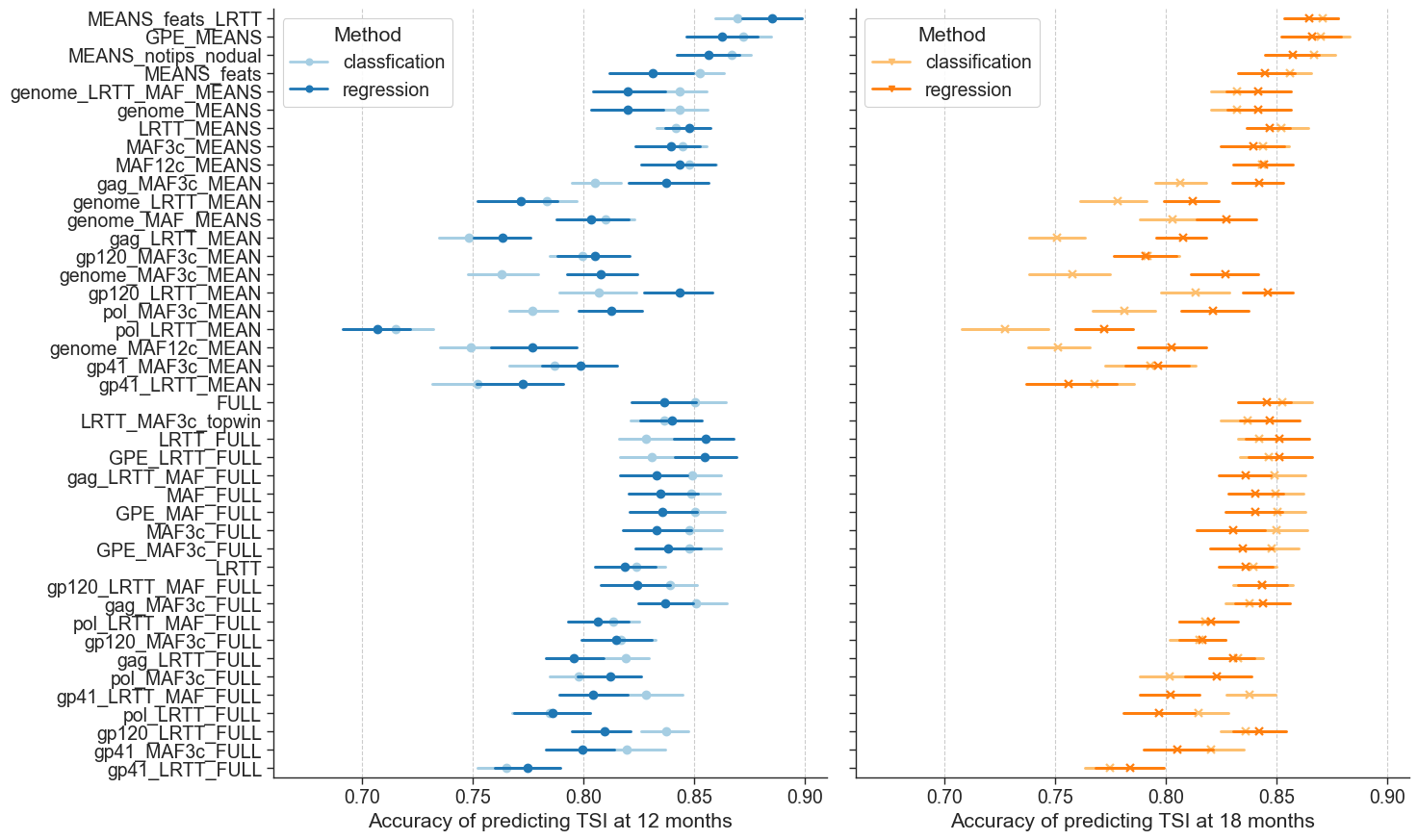


**B**


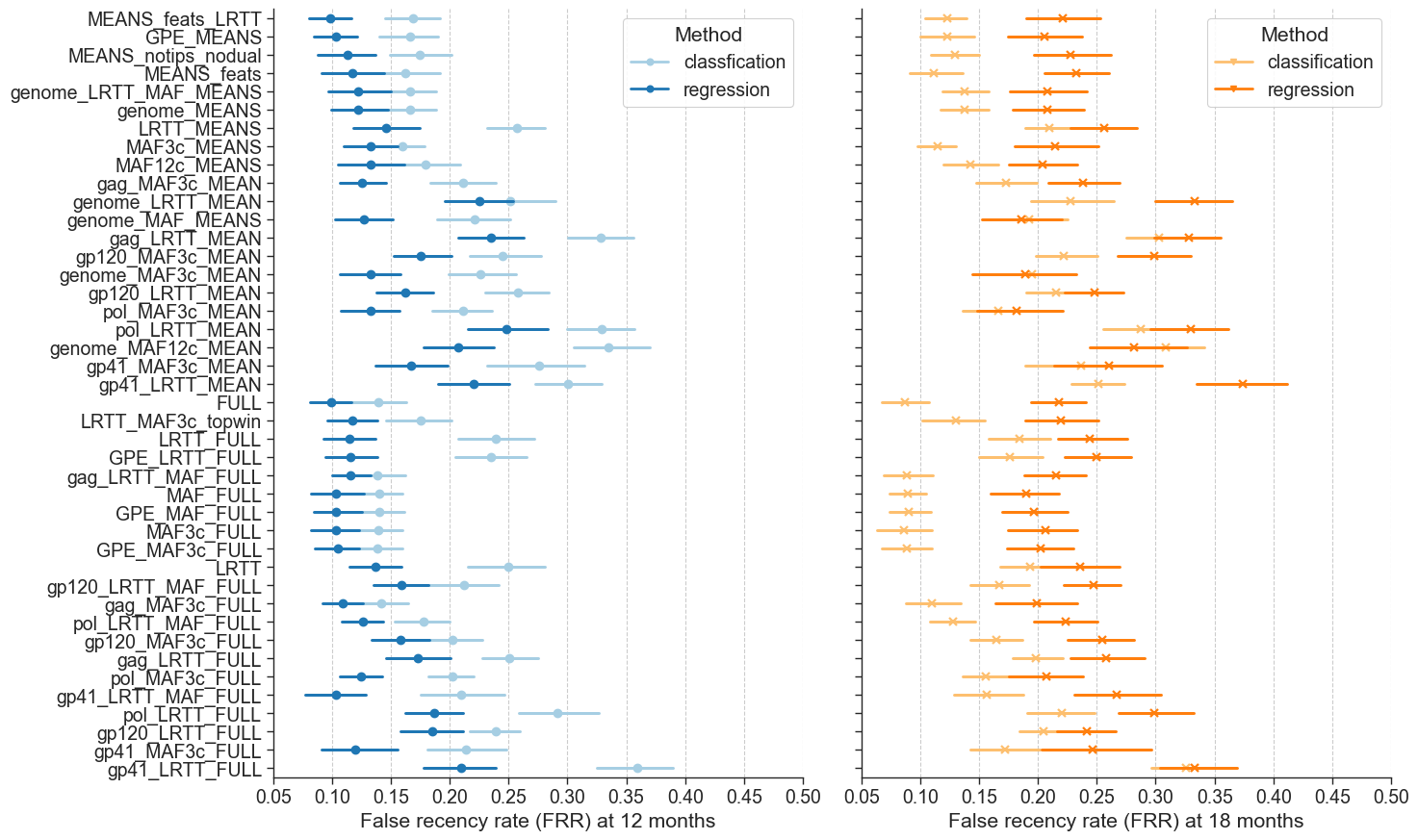


Fig. S7.

**Accuracy and False Recency Rate (FRR) of regression and classification models.** Points show mean score; vertical lines indicate 95% CI over 20-fold cross-validation. A. Accuracy was calculated as the proportion of samples correctly predicted at a threshold of 12 months (left panel) or 18 months (right panel), out of the total number of samples. B: False recency rate was calculated as the proportion of non-recent samples (with TSI above threshold) incorrectly classified as being recent with TSI < threshold at either 12 months (left panel) or 18 months (right panel).

**
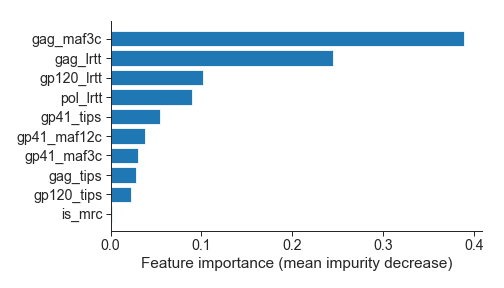
**

Fig. S8.

**Feature importances (mean decrease in impurity among 1000 decision trees) for feature set MEANS_feats_LRTT.** Contribution of each feature (proportional to variance explained) for the ten features in the best-performing regression models. Feature importances were extracted from the random forest model feature_importances_ attribute, computed within scikit-learn as the mean of accumulation of the impurity decrease within each tree.


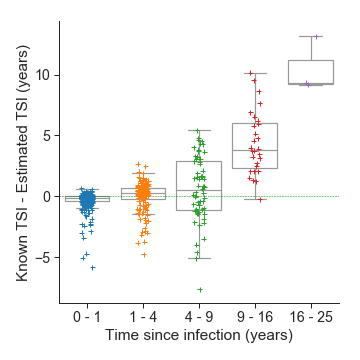


Fig. S9.

**Model bias is low for infections <9 years.** Boxplots show the median and quartiles for each TSI category; whiskers extend to 1.5 IQR. The difference between known and estimated TSI increases for non-recent infections. TSI for recent infections (under 1 year) tends to be slightly overestimated, while TSI for long-term infections is increasingly underestimated.

|  |  |  | **Regression Method** | | |
| --- | --- | --- | --- | --- | --- |
| **Feature set** | **Predictors** | **Number of predictors** | **Linear OLS** | **Gradient boosted** | **Random forest** |
| **genome_LRTT_MEAN** | is_mrc, genome_lrtt | 2 | 0.409 | 0.446 | 0.455 |
| **genome_MAF12c_MEAN** | is_mrc, genome_maf12c | 2 | 0.304 | 0.185 | 0.218 |
| **genome_MAF3c_MEAN** | is_mrc, genome_maf3c | 2 | 0.219 | 0.248 | 0.322 |
| **gag_LRTT_MEAN** | is_mrc, gag_lrtt | 2 | 0.377 | 0.412 | 0.412 |
| **gag_MAF3c_MEAN** | is_mrc, gag_maf3c | 2 | -0.038 | 0.388 | 0.452 |
| **gp120_LRTT_MEAN** | is_mrc, gp120_lrtt | 2 | 0.335 | 0.312 | 0.323 |
| **gp120_MAF3c_MEAN** | is_mrc, gp120_maf3c | 2 | 0.252 | 0.341 | 0.349 |
| **gp41_LRTT_MEAN** | is_mrc, gp41_lrtt | 2 | 0.294 | 0.221 | 0.229 |
| **gp41_MAF3c_MEAN** | is_mrc, gp41_maf3c | 2 | 0.107 | 0.211 | 0.226 |
| **pol_LRTT_MEAN** | is_mrc, pol_lrtt | 2 | 0.334 | 0.290 | 0.302 |
| **pol_MAF3c_MEAN** | is_mrc, pol_maf3c | 2 | 0.051 | 0.291 | 0.310 |
| **genome_MAF_MEANS** | is_mrc, genome_maf3c, genome_maf12c | 3 | 0.206 | 0.248 | 0.329 |
| **LRTT_MEANS** | is_mrc, gag_lrtt, pol_lrtt, gp120_lrtt, gp41_lrtt, genome_lrtt | 6 | 0.406 | 0.517 | 0.558 |
| **MAF12c_MEANS** | is_mrc, gag_maf12c, pol_maf12c, gp120_maf12c, gp41_maf12c, genome_maf12c | 6 | 0.267 | 0.375 | 0.434 |
| **MAF3c_MEANS** | is_mrc, gag_maf3c, pol_maf3c, gp120_maf3c, gp41_maf3c, genome_maf3c | 6 | 0.060 | 0.449 | 0.463 |
| **genome_LRTT_MAF_MEANS** | is_mrc, genome_tips, genome_dual, genome_lrtt, genome_maf3c, genome_maf12c | 6 | 0.522 | 0.500 | 0.532 |
| **genome_MEANS** | is_mrc, genome_tips, genome_dual, genome_lrtt, genome_maf3c, genome_maf12c | 6 | 0.522 | 0.500 | 0.532 |
| **MEANS_feats** | is_mrc, genome_lrtt, genome_maf3c, gag_lrtt, gag_maf3c, pol_lrtt, gp41_lrtt, pol_maf12c | 8 | 0.383 | 0.594 | 0.598 |
| **MEANS_feats_LRTT** | is_mrc, gag_lrtt, pol_lrtt, gp120_lrtt, gag_maf3c, gp41_maf3c, gp41_maf12c, gag_tips, gp41_tips, gp120_tips | 10 | 0.489 | 0.640 | 0.650 |
| **MEANS_notips_nodual** | is_mrc, gag_lrtt, gag_maf3c, gag_maf12c, pol_lrtt, pol_maf3c, pol_maf12c, gp120_lrtt, gp120_maf3c, gp120_maf12c, gp41_lrtt, gp41_maf3c, gp41_maf12c, genome_lrtt, genome_maf3c, genome_maf12c | 16 | 0.320 | 0.614 | 0.630 |
| **GPE_MEANS** | is_mrc, genome_tips, genome_dual, gag_lrtt, gag_tips, gag_maf3c, gag_maf12c, pol_lrtt, pol_tips, pol_maf3c, pol_maf12c, gp120_lrtt, gp120_tips, gp120_maf3c, gp120_maf12c, gp41_lrtt, gp41_tips, gp41_maf3c, gp41_maf12c | 19 | 0.452 | 0.636 | 0.638 |

Table S1.

Cross-validated scores (mean r^2^ values in 10 folds) for different sets of LRTT, MAF3c and/or MAF12c feature combinations, computed using ordinary least squares (OLS), gradient boosted and random forest regression.

| **Months** | **Accuracy** | **False recency rate** | **True recency rate** |
| --- | --- | --- | --- |
| **3** | 93.0 | 2.2 | 49.7 |
| **6** | 86.6 | 4.7 | 65.9 |
| **12** | 80.1 | 11.6 | 74.0 |
| **18** | 86.1 | 10.3 | 84.6 |
| **24** | 89.6 | 5.8 | 88.4 |

Table S2.

**Accuracy, false recency rate and true recency rate for simulated population data, with recency defined variously as infections occurring in the preceding 3, 6, 12, 18 and 24 months.** One thousand individuals were drawn from a population with an average interval of three years from infection to treatment.

| **Group 1** | **Group 2** | **Mean between-group difference** | **Lower** | **Upper** | **Reject** |
| --- | --- | --- | --- | --- | --- |
| **A1** | **B** | -0.0079 | -0.1575 | 0.1417 | FALSE |
| **A1** | **C** | 0.0188 | -0.104 | 0.1416 | FALSE |
| **A1** | **D** | -0.0015 | -0.1369 | 0.1339 | FALSE |
| **A1** | **Other** | -0.0251 | -0.17 | 0.1198 | FALSE |
| **B** | **C** | 0.0267 | -0.1095 | 0.1629 | FALSE |
| **B** | **D** | 0.0064 | -0.1413 | 0.154 | FALSE |
| **B** | **Other** | -0.0171 | -0.1736 | 0.1393 | FALSE |
| **C** | **D** | -0.0203 | -0.1407 | 0.1001 | FALSE |
| **C** | **Other** | -0.0438 | -0.1749 | 0.0872 | FALSE |
| **D** | **Other** | -0.0235 | -0.1664 | 0.1194 | FALSE |

Table S3.

**Tukey range test result for pairwise subtype comparisons of model bias.** Mean bias of model predictions for all samples, adjusted for time since infection, compared using the Tukey range test for pairwise comparison of means, as implemented in the statsmodels python library. None of the subtypes differed at p<0.05 (reject=False).
